# Evolution and losses of spines in slug caterpillars (Lepidoptera: Limacodidae)

**DOI:** 10.1002/ece3.5524

**Published:** 2019-08-05

**Authors:** Yu‐Chi Lin, Rung‐Juen Lin, Michael F. Braby, Yu‐Feng Hsu

**Affiliations:** ^1^ Department of Life Science National Taiwan Normal University Taipei Taiwan; ^2^ Division of Ecology and Evolution Research School of Biology The Australian National University Acton ACT Australia; ^3^ The Australian National Insect Collection National Research Collections Australia Canberra ACT Australia

**Keywords:** character evolution, molecular phylogeny, morpho‐chemical defense, Zygaenoidea

## Abstract

Larvae of the cosmopolitan family Limacodidae, commonly known as “slug” caterpillars, are well known because of the widespread occurrence of spines with urticating properties, a morpho‐chemical adaptive trait that has been demonstrated to protect the larvae from natural enemies. However, while most species are armed with rows of spines (“nettle” caterpillars), slug caterpillars are morphologically diverse with some species lacking spines and thus are nonstinging. It has been demonstrated that the evolution of spines in slug caterpillars may have a single origin and that this trait is possibly derived from nonstinging slug caterpillars, but these conclusions were based on limited sampling of mainly New World taxa; thus, the evolution of spines and other traits within the family remains unresolved. Here, we analyze morphological variation in slug caterpillars within an evolutionary framework to determine character evolution of spines with samples from Asia, Australia, North America, and South America. The phylogeny of the Limacodidae was reconstructed based on a multigene dataset comprising five molecular markers (5.6 Kbp: COI, 28S, 18S, EF‐1α, and wingless) representing 45 species from 40 genera and eight outgroups. Based on this phylogeny, we infer that limacodids evolved from a common ancestor in which the larval type possessed spines, and then slug caterpillars without spines evolved independently multiple times in different continents. While larvae with spines are well adapted to avoiding generalist predators, our results imply that larvae without spines may be suited to different ecological niches. Systematic relationships of our dataset indicate six major lineages, several of which have not previously been identified.

## 1 INTRODUCTION

When similar phenotypes occur in a broadly distributed taxonomic clade, it may result from one or more processes, including inheritance from a common ancestor (homology), adaptation to similar local environments, shared constraints, and random genetic drift (homoplasy) (Jacobs et al., 2013; Losos, 2011; Stewart, 2007). Homoplasy, the phenotypic similarity resulting from independent evolution, is an important and common phenomenon, and it may arise in three different ways (Brooks, 1996; Hall, 2007; Lankester, 1870; Losos, 2011; McGhee, 2011; Meyer, 1999; Stayton, 2008; Wake, 1996). Firstly, it may reveal that natural selection produces optimal solutions to repeated problems posed by similar environments (Gordon & Notar, 2015; Larson & Losos, 1996; Losos, 2011; McGhee, 2011; Wake, Wake, & Specht, 2011). For example, mimicry is a form of homoplasy in which one species (the mimic) independently evolves a similar phenotype to a harmful or distasteful species (the model) to avoid predation (McGhee, 2011; Rettenmeyer, 1970; Sherratt, 2008; Symula, Schulte, & Summers, 2001). Secondly, homoplasy may reveal that genetic or developmental constraints limit the generation of phenotypic variations (Brakefield, 2006; Losos, 2011; McGhee, 2011; McKitrick, 1993; Powell, 2007; Uller, Moczek, Watson, Brakefield, & Laland, 2018; Wake, 1991; Wake et al., 2011). For example, digit loss in amphibians has occurred repeatedly during their evolutionary history, but the adaptive significance is not clear and it may simply represent developmental constraints (Alberch & Gale, 1985; Amundson, 2001; Autumn et al., 2002; Lamb & Beamer, 2012; Reeve & Sherman, 1993; Wake, 1991). Thirdly, homoplasy may result from random genetic drift (Jacobs et al., 2013; Jacobs, Mutumi, Maluleke, & Webala, 2016; Stayton, 2008). For example, homoplasy of morphology and echolocation frequency in the bats, *Rhinolophus darling* and *R. damarensis*, may be the result of random genetic drift, after excluding adaptation to similar local environments and shared constraints (Jacobs et al., 2013, 2016). In order to recognize homoplasy, it is critical to distinguish synapomorphic traits from convergent traits, which can be achieved using a phylogenetic systematics approach (Eldredge & Cracraft, 1980; Gordon & Notar, 2015; Larson & Losos, 1996; Losos, 2011; McGhee, 2011; Wake et al., 2011).

Antipredator strategies occur in every biome of the world, implying that predation is a potent selective force and thus of immense ecological and evolutionary significance (Grimaldi & Engel, 2005; Murphy, Leahy, Williams, & Lill, 2010; Ruxton, Sherratt, & Speed, 2004). Spines are one kind of obvious antipredator strategy to avoid predation (Inbar & Lev‐Yadun, 2005), such as the spines on inflated pufferfish (Brainerd, 1994), sticklebacks (Gross, 1978; Hoogland, Morris, & Tinbergen, 1956; Reimchen, 1983), slug caterpillars of the moth family Limacodidae (Murphy et al., 2010) and those on spiny plants (Gowda, 1996; Hanley, Lamont, Fairbanks, & Rafferty, 2007; Lev‐Yadun, 2001). Spines are a common defense mechanism that have evolved independently (homoplasy) in aquatic and terrestrial ecosystems, indicating that the reappearance of this phenotype is highly adaptive. However, antipredator strategies may be secondarily lost due to various  factors, for example, due to the loss of predators or limited nutrients (Bell, Francis, & Havens, 1985; Giles, 1983; Larson, 1976; McNab, 1994; Whitwell et al., 2012). Thus, it may be difficult to distinguish whether similar phenotypes present in a broadly distributed taxonomic clade is due to gains or losses. Hence, integrating phenotypic variation and reconstructing the probable ancestral states within a phylogenetic framework can enhance our knowledge of how traits evolve and may provide insights into the evolutionary processes and selective pressures involved.

**Figure 1:**
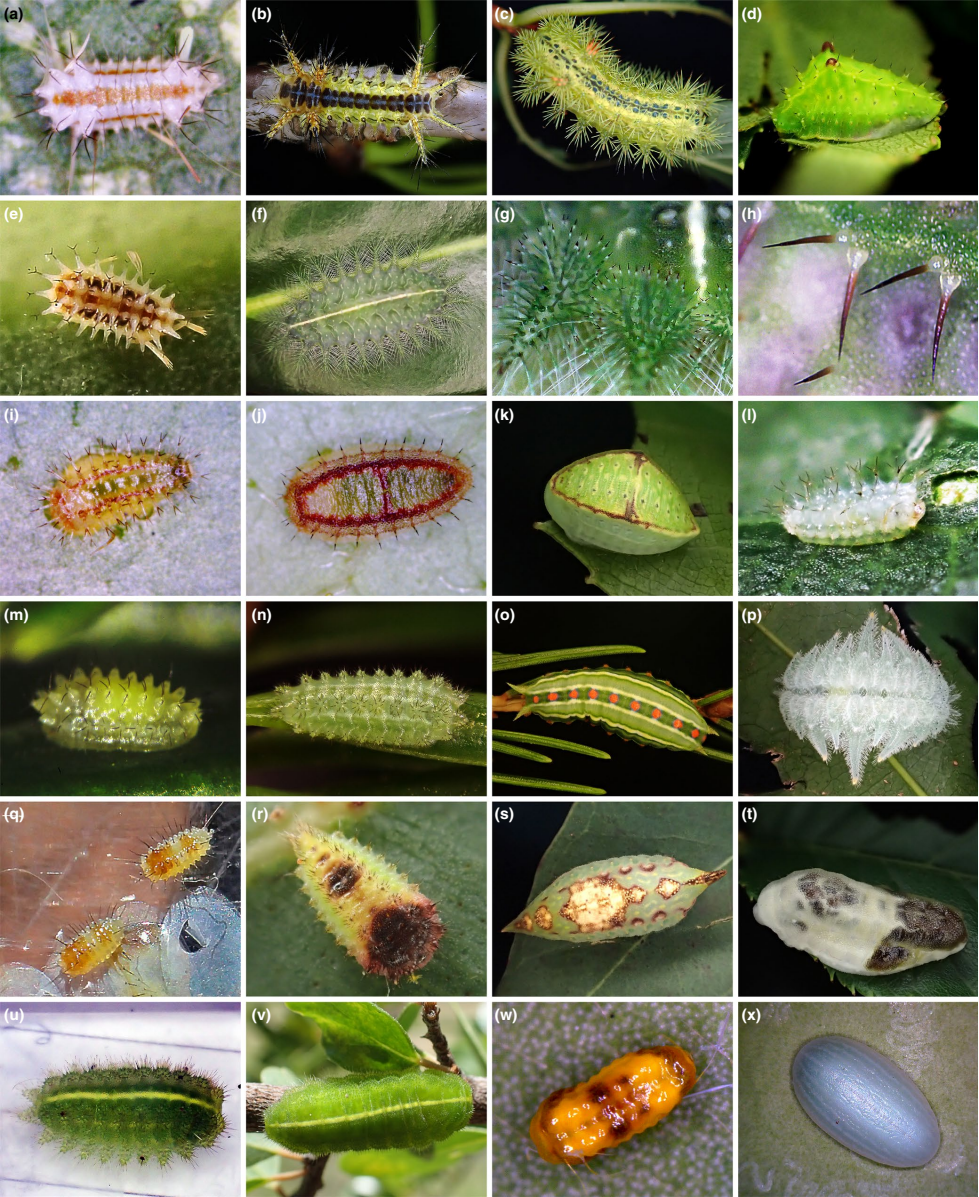
Different larval types of slug caterpillars in the Limacodidae with respect to the presence of spines: (a–c) first, early, and late instar of *Parasa consocia* (character state A: spines present after second instar); (d) late instar of *Microleon longipalpis* (character state A); (e–f) first and late instar larva of *Cania heppneri* (character state A); (g) spines on the late instar of *Cania heppneri*; (h) spines on the late instar of *Microleon longipalpis*; (i) first instar of *Demonarosa rufotessellata subrosea* (character state B: spines present after second instar but reduced in late instars); (j) second instar of *Demonarosa rufotessellata subrosea* with spines on the segments (character state B); (k) late instar of *Demonarosa rufotessellata subrosea* with almost all spines lost (character state B); (l) first instar of *Phrixolepia inouei* (character state D: spines absent but numerous setae present after second instar); (m) first instar of *Caiella pygmy* (character state B); (n) early instar of *Caiella pygmy* with spines (character state B); (o) late instar of *Caiella pygmy* with almost all spines reduced (character state B); (p) late instar of *Phrixolepia inouei* with numerous setae (character state D); (q) first instar of *Pseudanapaea transvestita* (character state B); (r) second instar of *Pseudanapaea transvestita* with spines (character state B); (s) late instar of *Pseudanapaea transvestita* with almost all spines reduced (character state B); (t) late instar larva of *Nagodopsis shirakiana* (character state C: spines absent in all instars); (u) early instar of *Ecnomoctena brachyopa* with spines (character state B); (v) late instar of *Ecnomoctena brachyopa* with almost all spines reduced (character state B); (w, x) first and late instar of *Altha melanopsis* (character state C)

Caterpillars play a major role in herbivory, but while feeding they are susceptible to attack from natural enemies (Reed, Grotan, Jenouvrier, Sather, & Visser, 2013). To protect themselves from predators and parasitoids, caterpillars have evolved a diverse array of antipredator strategies, including chemical, physiological, morphological, and behavioral responses (Greeney, Dyer, & Smilanich, 2012). Spines and setae in caterpillars are one kind of morphological–chemical adaptive response to avoid predation. At least 13 families of Lepidoptera, including the Limacodidae, have been recorded in which the caterpillars possess stinging (urticating) properties via spines and setae (Battisti, Holm, Fagrell, & Larsson, 2011; Hossler, 2010; Kano, 1977; Kawamoto & Kumada, 1984; Mullen, 2009). Spines and setae may injure predators or impose a cost in terms of increased handling time (Murphy et al., 2010; Petrucco Toffolo et al., 2014; Sugiura & Yamazaki, 2014). The Limacodidae, containing more than 1,650 species (Nieukerken et al., 2011), occur in all zoogeographic regions of the world (Cock, Godfray, & Holloway, 1987; Epstein, Geertsema, Naumann, & Tarmann, 1999), and their slug caterpillars are morphologically diverse (Figure 1) (Cock et al., 1987; Murphy, Lill, & Epstein, 2011). Three main types of slug caterpillars have been distinguished among late instars: (a) larvae armed with rows of spines (“nettle” caterpillars); (b) larvae with no spines on a relatively smooth surface (“gelatine” caterpillars); and (c) larvae with many fine setae on tubercles that can be detached (“monkey” slug caterpillars) (Cock et al., 1987; Dyar, 1896, 1907; Zaspel, Weller, & Epstein, 2016). Nettle caterpillars and gelatine caterpillars are almost distributed globally, whereas monkey slugs are rare, occurring in low abundance and being geographically restricted to Asia and the New World. The majority of limacodid larvae are nettle caterpillars, which are armed with spines that are well known to inflict stings (Hossler, 2010; Kawamoto, 1978; Murphy et al., 2010; Walker, 2018; Zaspel et al., 2016).

Murphy et al. (2010) presented evidence that spines do indeed protect slug caterpillars from generalist predators. Cock et al. (1987) presented a hypothesis that nonstinging types of slug caterpillars evolved from nettle caterpillars. However, the first detailed phylogenetic study of Limacodidae by Zaspel et al. (2016) suggested that (a) nettle caterpillars are a monophyletic group; (b) gelatine caterpillars are a monophyletic group; and (c) nettle caterpillars are derived from gelatine caterpillars. Because the study of Zaspel et al. (2016) was based on mainly New World taxa, the results may be derived from in situ diversification or independent colonization. Thus, it is uncertain if the evolutionary pattern of slug caterpillars is the same after including samples from different zoogeographic regions of the world. It is also unclear whether the existence or loss of spines in slug caterpillars has evolved once or has evolved repeatedly and independently in different lineages and/or in different continents.

When spines are present, they may be derived from a common ancestor or the result of homoplasy. Furthermore, because antipredator features may be secondarily lost, nettle, and gelatine caterpillars may be the result of multiple gains or losses of spines. Hence, our objectives were as follows: (a) to reconstruct a well‐supported phylogeny of the Limacodidae using a multigene dataset and (b) to trace the evolution of spines by optimizing character states of slug caterpillars with and without spines on this phylogenetic framework. We also comment on the systematic relationships of the Limacodidae. Most of the taxa included in this study were reared from samples collected from Asia, but we also include material from Australia, North America, and South America.

## 2 MATERIALS AND METHODS

### 2.1 Phylogenetic reconstruction

#### 2.1.1 Taxon sampling

A total of 53 samples representing 45 ingroup species and 40 genera of the Limacodidae from Asia, Australia, North America, and South America were included for DNA extraction and phylogenetic analysis. We used eight outgroup species, including exemplars from Dalceridae, Lacturidae, Megalopygidae, Phaudidae, and Zygaenidae belonging to the superfamily Zygaenoidea. Among the five outgroup families, Dalceridae and Phaudidae are the most closely related families to the Limacodidae according to previous higher‐level phylogenetic studies (Epstein, 1996; Niehuis, Naumann, & Mishof, 2006; Regier et al., 2013). Most of the samples were collected and reared by the authors. Specimens were identified by DNA barcoding with BOLD Systems (Ratnasingham & Hebert, 2007) (http://www.barcodinglife.org/) or BLAST (Johnson et al., 2008) (http://www.ncbi.nlm.nih.gov/BLAST), and by morphological traits on Catalogue of Life in Taiwan (Biodiversity Research Center, 2018) and CSIRO‐Australian Moths Online (CSIRO, 2018). All exemplar species for this study are listed in Table 1.

**Table 1:** List of species used in the phylogenetic analysis for this study, their broad geographical distribution, larval character states A–D (A = spines present after second instar; B = spines present after second instar but reduced in late instars; C = spines and setae absent in all instars; D = spines absent but numerous setae present after second instar), and GenBank accession numbers

Taxon	Geographical region	Character state	GenBank accession number				
			COI	28S	18S	EF‐1α	Wingless
Ingroup							
Limacodidae							
*Altha melanopsis*	Asia	C	MK128255	MK128153	MK128204	MK128308	MK128360
*Anaxidia lozogramma*	Australia	A	MK128292	MK128190	MK128241	MK128345	MK128397
*Apoda y‐inversa*	North America	B	MK128294	MK128192	MK128243	MK128347	MK128399
*Belippa horrida*	Asia	C	MK128259	MK128157	MK128208	MK128312	MK128364
*Birthamoides plagioscia*	Australia	Unknown	MK128287	MK128185	MK128236	MK128340	MK128392
*Birthamula rufa*	Asia	A	MK128261	MK128159	MK128210	MK128314	MK128366
*Caiella pygmy*	Asia	B	MK128278	MK128176	MK128227	MK128331	MK128383
*Calcarifera ordinata*	Australia	A	MK128285	MK128183	MK128234	MK128338	MK128390
*Cania heppneri*	Asia	A	MK128263	MK128161	MK128212	MK128316	MK128368
*Ceratonema apodina*	Asia	B	MK128262	MK128160	MK128211	MK128315	MK128367
*Chalcocelis albiguttatus*	Australia	C	MK128288	MK128186	MK128237	MK128341	MK128393
*Chalcoscelides castaneipars*	Asia	C	MK128257	MK128155	MK128206	MK128310	MK128362
*Demonarosa rufotessellata subrosea*	Asia	B	MK128271	MK128169	MK128220	MK128324	MK128376
*Doratifera quadriguttata*	Australia	A	MK128286	MK128184	MK128235	MK128339	MK128391
*Doratifera vulnerans*	Australia	A	MK128290	MK128188	MK128239	MK128343	MK128395
*Ecnomoctena brachyopa*	Australia	A	MK128289	MK128187	MK128238	MK128342	MK128394
*Flavinarosa obscura*	Asia	A	MK128272	MK128170	MK128221	MK128325	MK128377
*Griseothosea fasciata*	Asia	A	MK128253	MK128151	MK128202	MK128306	MK128358
*Hampsonella arizana*	Asia	B	MK128254	MK128152	MK128203	MK128307	MK128359
*Isa textula*	North America	A	MK128296	KR068974	KR068941	MK128349	MK128401
*Isochaetes* sp.	South America	D	MK128303	MK128199	MK128250	MK128355	MK128408
*Microleon longipalpis*	Asia	A	MK128277	MK128175	MK128226	MK128330	MK128382
*Monema rubriceps*	Asia	A	MK128266	MK128164	MK128215	MK128319	MK128371
*Nagodopsis shirakiana*	Asia	C	MK128276	MK128174	MK128225	MK128329	MK128381
*Narosa nigrisigna*	Asia	B	MK128265	MK128163	MK128214	MK128318	MK128370
*Natada nasoni*	North America	A	MK128295	KR068981	KR068948	MK128348	MK128400
*Orthocraspeda furva*	Asia	A	MK128267	MK128165	MK128216	MK128320	MK128372
*Parasa consocia*	Asia	A	MK128258	MK128156	MK128207	MK128311	MK128363
*Parasa pastoralis*	Asia	A	MK128281	MK128179	MK128230	MK128334	MK128386
*Parasa shirakii*	Asia	A	MK128269	MK128167	MK128218	MK128322	MK128374
*Parasa sinica*	Asia	A	MK128279	MK128177	MK128228	MK128332	MK128384
*Phlossa conjuncta*	Asia	A	MK128256	MK128154	MK128205	MK128309	MK128361
*Phrixolepia inouei*	Asia	D	MK128274	MK128172	MK128223	MK128327	MK128379
*Pseudanapaea transvestita*	Australia	B	MK128291	MK128189	MK128240	MK128344	MK128396
*Quasinarosa corusca*	Asia	B	MK128273	MK128171	MK128222	MK128326	MK128378
*Sansarea formosana*	Asia	B	MK128268	MK128166	MK128217	MK128321	MK128373
*Scopelodes contractus*	Asia	A	MK128252	MK128150	MK128201	MK128305	MK128357
*Setora baibarana*	Asia	A	MK128284	MK128182	MK128233	MK128337	MK128389
*Setora postornata*	Asia	A	MK128260	MK128158	MK128209	MK128313	MK128365
*Spatulifimbria castaneiceps opprimata*	Asia	A	MK128280	MK128178	MK128229	MK128333	MK128385
*Thosea sinensis*	Asia	B	MK128264	MK128162	MK128213	MK128317	MK128369
*Trichogyia limacodiformis*	Asia	A	MK128283	MK128181	MK128232	MK128336	MK128388
*Vanlangia castanea*	Asia	A	MK128275	MK128173	MK128224	MK128328	MK128380
Unplaced genus sp. 1	Asia	A	MK128282	MK128180	MK128231	MK128335	MK128387
Unplaced genus sp. 2	Asia	D	MK128293	MK128191	MK128242	MK128346	MK128398
Outgroup							
Dalceridae							
*Acraga melinda*	South America	Unknown	MK128301	MK128197	MK128248	MK128353	MK128406
Lacturidae							
*Eustixis sapotearum*	Australia	B or C	MK128300	MK128196	MK128247		MK128405
Megalopygidae							
*Megalopyge opercularis*	North America	A	MK128297	MK128193	MK128244	MK128350	MK128402
*Norape ovina*	North America	A	MK128299	MK128195	MK128246	MK128352	MK128404
Phaudidae							
*Phauda mimica*	Asia	C	MK128270	MK128168	MK128219	MK128323	MK128375
*Phauda* sp.	Asia	C	MK128302	MK128198	MK128249	MK128354	MK128407
Zygaenidae							
*Clelea formosana*	Asia	Unknown	MK128298	MK128194	MK128245	MK128351	MK128403
*Erasmia pulchella hobsoni*	Asia	A	MK128251	MK128149	MK128200	MK128304	MK128356

Note

Taxa are listed alphabetically.

#### 2.1.2 Molecular data

Total genomic DNA was extracted from 1 to 3 legs of each specimen using a commercial DNA extraction kit (Gentra Puregene Tissue kit, Qiagen) following the manufacturer's protocol. The polymerase chain reaction (PCR) was used to amplify the following five gene fragments: cytochrome oxidase subunit I (COI), D2 region of the 28S ribosomal sequence, 18S ribosomal sequence, elongation factor‐1 alpha (EF‐1α), and partial sequences of the wingless gene. The first mentioned fragment is encoded in the mitochondrial genome, whereas the remaining four markers are part of the nuclear genome. These genetic markers are phylogenetically informative and commonly used for resolving the systematics of the Lepidoptera (Chalwatzis, Baur, Stetzer, Kinzelbach, & Zimmermann, 1995; Lee & Brown, 2008; Lo et al., 2015; Mutanen, Wahlberg, & Kaila, 2010; Niehuis et al., 2006; Regier et al., 2013, 2009; Simon et al., 1994; Wahlberg & Wheat, 2008; Zaspel et al., 2016). A list of primers used for generating sequence data from the targeted loci is given in Table 2. Most of the primers have been published in previous studies, but several new primers for 18S ribosomal sequence and wingless were designed for this study. In addition, four sequences (18S and 28S for both *Apoda y‐inversa* and *Natada nasoni*) were downloaded from GenBank NCBI (https://www.ncbi.nlm.nih.gov/genbank/).

**Table 2:** List of primers used for generating sequence data for the five genetic markers

Marker	Primer Name	Primer sequence	Reference
COI	Pat	TCC AAT GCA CTA ATC TGC CAT ATT A	Simon et al. (1994)
	Jerry	CAA CAT TTA TTT TGA TTT TTT GG	Simon et al. (1994)
	Ron	GGA TCA CCT GAT ATA GCA TTC CC	Simon et al. (1994)
	Nancy	CCC GGT AAA ATT AAA ATA TAA ACT TC	Simon et al. (1994)
	K698	TAC AAT TTA TCG CCT AAA CTT CAG CC	Simon et al. (1994)
	K808	TGG AGG GTA TAC TGT TCA ACC	Simon et al. (1994)
28S	28S‐f1	GAG TAC GTG AAA CCG TTC AG	Lee and Brown (2008)
	28S‐r1	CTG ACC AGG CAT AGT TCA C	Lee and Brown (2008)
18S	18S‐f1	TAC CTG GTG GAT CCT GCC AGT	Chalwatzis et al. (1995)
	18S‐f2	GAT ACG GGA CTC TTA CGA GG	Niehuis et al. (2006)
	18S‐f3	GGT GTT TTC ATC AAT CAA G	Niehuis et al. (2006)
	18S‐f4	TCC GAT AAC GAA CGA GAC TC	Niehuis et al. (2006)
	18S‐r1	TAA CCG CAA CAA CTT TAA T	DeSalle, Gatesy, Wheeler, and Grimaldi (1992)
	18S‐r2	GCT AGA TGA CAT TTT TAC GG	Niehuis et al. (2006)
	18S‐r3	CGC CGG TCC CTC TAA GAA G	Niehuis et al. (2006)
	18S‐r4	TAA TGA TCC TTC TGC AGG TTC	Chalwatzis et al. (1995)
	18S‐80F	AAG GCG ATA CCG CGA ATG GCT	This study
	18S‐858R	CAG CAT TTT GAG CCC GCT TTG	This study
EF‐1α	Starsky	CAC ATY AAC ATT GTC GTS ATY GG	Cho et al. (1995)
	Luke	CAT RTT GTC KCC GTG CCA KCC	Cho et al. (1995)
	Cho	GTC ACC ATC ATY GAC GC	Reed and Sperling (1999)
	Verdi	GAT ACC AGT CTC AAC TCT TCC	Nazari, Zakharov, and Sperling (2007)
	EF51.9	CAR GAC GTA TAC AAA ATC GG	Cho et al. (1995)
	EFrcM4	ACA GCV ACK GTY TGY CTC ATR TC	Cho et al. (1995)
Wingless	LepWg1	GAR TGY AAR TGY CAY GGY ATG TCT GG	Brower and DeSalle (1998)
	LepWg2	ACT ICG CAR CAC CAR TGG AAT GTR CA	Brower and DeSalle (1998)
	wg‐lim2F	GTG AAG ACY TGC TGG ATG AGG CT	This study
	wg‐lim425R	CCA ATG GAA TGT RCA GTT GCA	This study

The following PCR settings were adopted: 4 min at 94°C, followed by 40 cycles of 30 s at 94°C, 30 s at 60°C, and 40–60 s at 72°C. The final elongation step was continued for 10 min at 72°C and stopped at 4°C. If the above conditions failed, we amplified the fragments using a touchdown method: 4 min at 94°C, followed by 10 cycles of 30 s at 94°C, 30 s at 62°C decreasing 1°C each cycle, 40–60 s at 72°C and then followed by 35 cycles of 30 s at 94°C, 30 s at 52°C, and 40–60 s at 72°C. The final elongation step was continued for 10 min at 72°C and stopped at 4°C. The PCR products were conducted on agarose gel electrophoresis to verify successful amplification. Purified PCR products were sequenced with dye‐labeled terminators, and the dye‐labeled DNA fragments were read on ABI 3730XL Analyzer (Applied Biosystems).

#### 2.1.3 Phylogenetic analyses

The DNA sequences were checked and assembled with Sequencher 4.8 (GENCODE). The resulting multiple sequence alignments were achieved by MUSCLE (Edgar, 2004) implemented in MEGA (version 6) (Tamura, Stecher, Peterson, Filipski, & Kumar, 2013) and then adjusted manually by eye. Phylogenetic analyses were performed on the combined dataset of the five concatenated gene sequences. The combined dataset was allocated to 11 subsets with respect to the five gene fragments and to codon positions of protein‐coding genes; the best‐fit substitution model and subset partitions were then evaluated by PartitionFinder (version 1.1.1) (Lanfear, Calcott, Ho, & Guindon, 2012). Maximum likelihood (ML) and partitioned Bayesian Inference (BI) analyses were implemented separately by RAxML‐HPC BlackBox (version 8.2.9) (Stamatakis, 2014) and MrBayes XSEDE (version 3.2.6) (Ronquist & Huelsenbeck, 2003) on CIPRES (http://www.phylo.org/portal2/) (Miller, Pfeiffer, & Schwartz, 2010).

### 2.2 Character evolution

#### 2.2.1 Larval morphology

We collected eggs and larvae for most species to record larval character states. Some eggs were obtained from females collected from light traps, while other eggs and larvae were collected directly from the field. Eggs and larvae were brought back to the laboratory and assigned rearing records, adopting the system used by Powell and De Benedictis (1995). Each collection was labeled according to the collecting year and month, for example, 05G2 refers the second collection in July 2005 (this system employs alphabetical letters to represent months, e.g., G = July). Larvae were reared in plastic containers (150 mm × 80 mm × 45 mm). Vouchers are deposited in the Department of Life Sciences, National Taiwan Normal University (NTNU), Taipei.

#### 2.2.2 Coding of spines

Spines are composed of multiple cells; they involve poison‐secreting cells and neural cells (Battisti et al., 2011; Hossler, 2010; Kano, 1977). Spines cause urtication because the poison contents can be released into the skin from the broken tip of the spine (Battisti et al., 2011; Hossler, 2010; Kano, 1977; Kawamoto & Kumada, 1984; Mullen, 2009).

Based on previous studies (Battisti et al., 2011; Epstein, 1996; Murphy et al., 2011; Zaspel et al., 2016) and extensive rearing by the authors in the present study, spines of limacodid larvae usually form on protuberances (Figure 1g,h), which change in size on different segments, different instars, and among different species. For example, in *Parasa consocia* (Figure 1b,c) some protuberances are longer in early instars than in late instars. Thus, we focused mainly on the presence or absence of spines in the larval developmental stages. For the three main types of limacodid larvae, we recognized four character states based on the presence or absence of spines and setae throughout the entire larval developmental stage, as follows:

State A: Spines present after the second instar (Figure 1b–d,f); a few setae are present on pairs of protuberances on each segment in the first instar (Figure 1a,e).
State B: Spines present after the second instar (Figure 1j,n,r,u), but almost all spines are lost or reduced in late instars (Figure 1k,o,s,v); when the spines are reduced, they are tiny and vestigial (Figure 1v). A few setae are present on pairs of protuberances on each segment in the first instar (Figure 1i,m,q).
State C: Spines absent in all instars (Figure 1t,w,x). Further, the setae in the first instar are also vestigial, such as *Belippa horrida* (Epstein, 1996).
State D: Spines absent; numerous setae are present on tubercles, which can be pulled off after the second instar (Figure 1p); a few setae are present on pairs of protuberances on each segment in the first instar (Figure 1l).

#### 2.2.3 Character evolution analyses

The character evolution of larval spine variation was reconstructed on the maximum clade credibility tree using the Mk1 evolutionary model as implemented in Mesquite (version 3.2) (Maddison & Maddison, 2017).

## 3 RESULTS

### 3.1 Phylogenetic patterns

**Figure 2:**
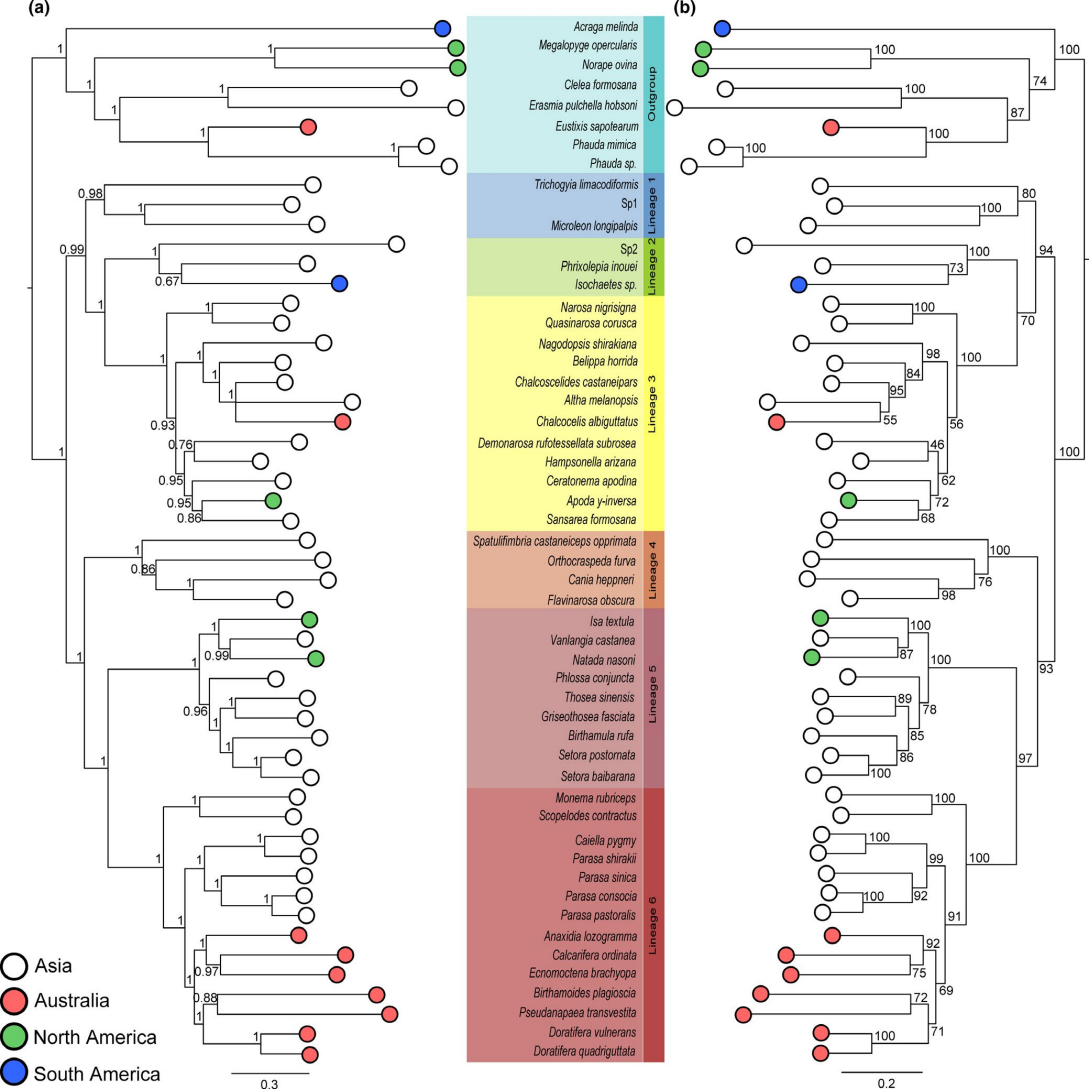
Phylogenetic trees of the Limacodidae based on the combined dataset constructed with: (a) partitioned Bayesian Inference; (b) partitioned Maximum Likelihood using the GTR + Γ+I substitution model. Branch lengths are proportional to inferred nucleotide substitutions, with values above nodes representing posterior probabilities (a) and ML bootstraps (b). Optimal topologies recovered by BI and ML were congruent. Six major lineages were recovered, which are indicated by different colors. Zoogeographic regions are represented in different colors on terminals, as per legend

The aligned sequences consisted of a total of 5,648 bp from 53 taxa, corresponding to the combinations of 1,510 bp COI, 674 bp 28S rRNA, 1865 bp 18S rRNA, 1,230 bp EF‐1α, and 369 bp wingless. The optimal topologies reconstructed by partitioned ML and Bayesian (BI) analyses were identical (Figure 2). Both ML and BI analyses strongly supported the monophyly of Limacodidae (ML bootstrap = 100%; Posterior probability = 1).

Within the inferred phylogenetic tree of the Limacodidae, six major clades (lineages 1–6) were identified with strong support (ML bootstrap = 100%; Posterior probability = 1 for lineages 2–6) and typically long basal branches (stems) (Figure 2). These clades fell into two reciprocally monophyletic groups, with lineages 1–3 sister to lineages 4–6. Lineage 1 with good support (ML bootstrap = 80%; Posterior probability = 0.98) included only nettle caterpillars from Asia. Lineage 2 included all hairy slug caterpillars from Asia and South America. The hairy slug caterpillars of lineage 2 were sister to lineage 3, which comprised gelatine caterpillars from Asia, North America, and Australia. Lineage 4 included nettle caterpillars from Asia, whereas lineage 5 included nettle caterpillars from both Asia and North America. Lineage 6 included mostly nettle caterpillars from Asia and Australia, but also three taxa in which spines were reduced: *Caiella pygmy* from Asia, and *Ecnomoctena brachyopa* and *Pseudanapaea transvestita* from Australia.

### 3.2 Character evolution of spines

**Figure 3:**
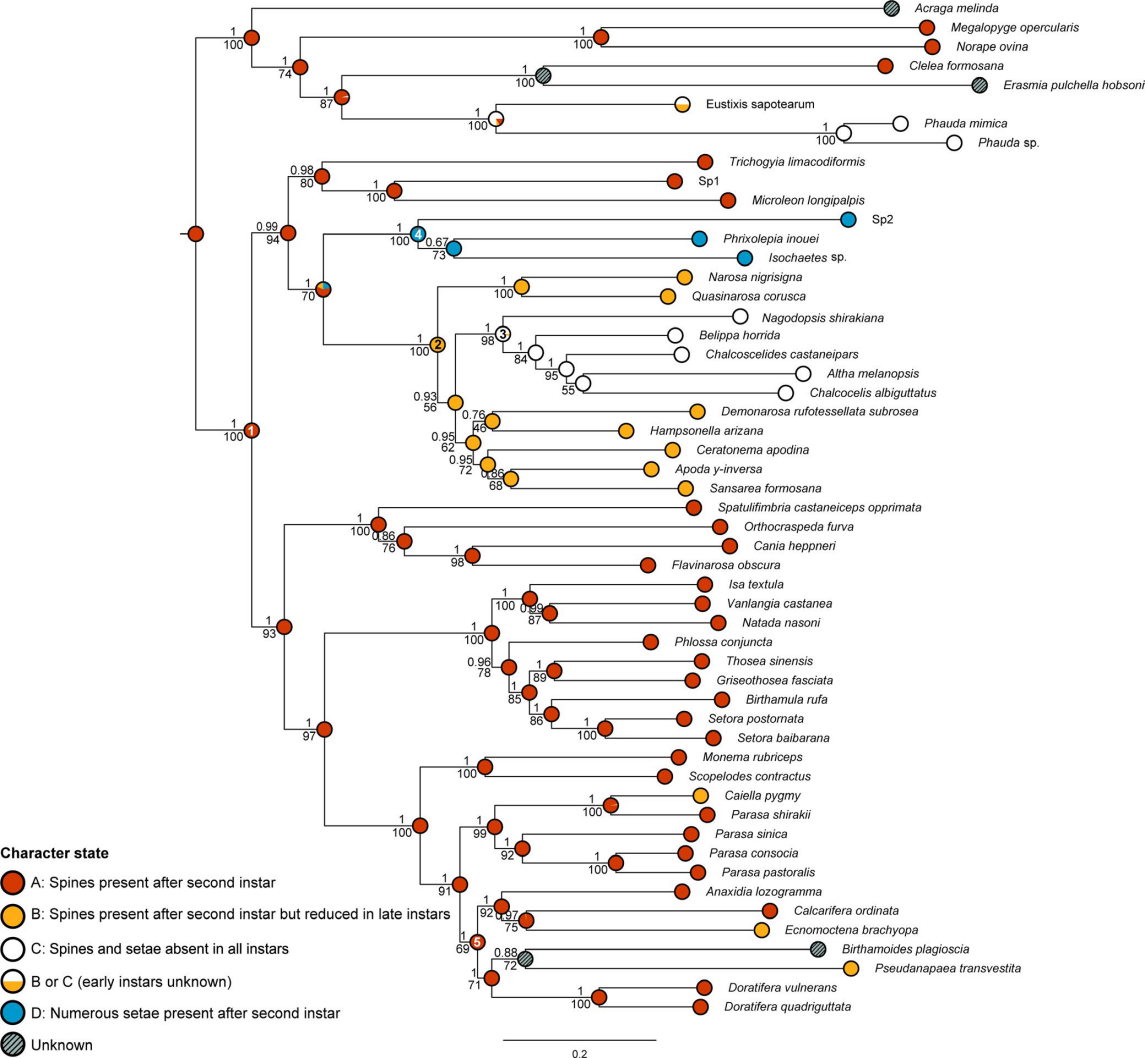
Phylogenetic tree of the Limacodidae constructed using partitioned Maximum Likelihood, with bootstrap values below branches and posterior probabilities above. Character state reconstruction for spines was carried out using Maximum Likelihood (Mesquite). The proportional likelihoods of the different character states in the ancestral reconstructions are indicated by the area red/yellow/white/blue in each pie diagram (A = red for spines present after second instar; B = yellow for spines present after second instar but reduced in late instars; C = white for spines and setae absent in all instars; D = blue for spines absent but numerous setae present after second instar). Node 1: proportional likelihood of character state A = 0.999. Node 2: proportional likelihood of character state B = 0.987. Node 3: proportional likelihood of character state C = 0.964. Node 4: proportional likelihood of character state D = 0.958. Node 5: proportional likelihood of character state A = 0.999

The evolutionary reconstruction of spines in limacodid caterpillars indicated that the ancestral state was most likely larvae with spines present from second instar to final instar (character state A) (Figure 3, Node 1: proportional likelihood of character state A = 0.999). There were four separate transitions from this ancestral character state to spines lost or reduced in late instars (character state B), which evolved independently three times in lineage 6 and once in lineage 3 (Figure 3, Node 2: proportional likelihood of character state *B* = 0.987). There was a further transition from spines lost or reduced in late instars to spines absent in all instars (character state C) in lineage 3 (Figure 3, Node 3: proportional likelihood of character state *C* = 0.963). There was another transition from spines present after second instar (character state A) to spines absent but numerous setae present after second instar (character state D) in lineage 2 (Figure 3, Node 4: proportional likelihood of character state *D* = 0.958).

## 4 DISCUSSION

Our molecular study provides a robust phylogeny of the Limacodidae. The well‐supported phylogenetic framework allows us to reliably reconstruct the character evolution of spines throughout the entire larval stage, to test previous hypotheses regarding the evolution of slug caterpillars, and to infer the potential mechanisms of homoplasy in limacodids.

### 4.1 Character evolution and morphological homoplasy

According to the phylogeny reconstructed in this study, limacodids evolved from a common ancestor in which the larval type possessed spines from second instar to final instar (character state A), and then, spines were evolutionary lost or reduced in late instars (character state B) multiple times—at least on four occasions (Figure 3). Of the four independent transitions from the presence of spines to the absence or reduction of spines in late instars, two were in Asia (ancestor of lineage 3 and *Caiella pygmy* in lineage 6), and two were in Australia (*Ecnomoctena brachyopa* and *Pseudanapaea transvestita* in lineage 6). Thus, we infer that loss or reduction in spines is the result of homoplasy in these zoogeographic regions. Moreover, spines absent in all instars (character state C) evolved once from a common ancestor in which spines were lost or reduced in late instars (character state B), indicating a clear evolutionary progression in the loss of poisonous spines from nettle caterpillars to gelatine caterpillars. This pattern is consistent with Cock's (1987) hypothesis that nonstinging types of slug caterpillars evolved from nettle caterpillars. Although the pattern contrasts with the larval character evolution of Zaspel et al. (2016), it must be emphasized that branch support for many of the basal nodes in that phylogenetic study was low and hence ancestral reconstructions were at best preliminary.

Spines in the Limacodidae are considered to be an adaptive response to predation (Murphy et al., 2010). Our phylogeny indicates that this defense strategy evolved early in the origin of the family, and the trait is widespread across lineages 1 and 4–6 (Figure 3). Therefore, the independent losses of poisonous spines (homoplasy) raise the interesting question as to why have some larvae evolutionary lost their toxic antipredator mechanism? Gelatine caterpillars avoid predation through crypsis or masquerade, but it remains to be determined what mechanism may have driven this type of defense strategy. Here, we propose several potential mechanisms (hypotheses) for spine reduction in slug caterpillars.

The first hypothesis is that spines get lost or reduced because they confer no advantage below a certain size threshold. It has been demonstrated that defensive characters such as warning coloration are more effective when displayed in insects with large bodies (Forsman & Merilaita, 1999; Hossie, Skelhorn, Breinholt, Kawahara, & Sherratt, 2015). For example, defensive eyespots are effective in big caterpillars, but costly in small caterpillars, because they enhance detectability without providing a protective advantage in small caterpillars (Hossie et al., 2015). In tree‐feeding insects, avian predation risk increased with larger prey body size (Remmel, Davison, & Tammaru, 2011; Remmel & Tammaru, 2009). Therefore, slug caterpillars with small body size (e.g., *Quasinarosa corusca*) may be hard to detect, so that the cost of producing spines and toxins may be higher than the benefit of avoiding predation in smaller taxa.

The second hypothesis is that there has been a change in predator pressure. Predators (e.g., insectivorous birds) eat aposematic prey in a selective manner according to their levels of hunger and the presence of alternative prey (Cott, 1940; Ruxton et al., 2004). When limacodids expand their range or enter new adaptive zones, such as in low diversity biomes (e.g., high mountain or desert habitats), with potentially higher levels of predator pressure and less alternative prey, nettle caterpillars may be too obvious to survive and cryptic larvae without spines may be selected for.

The third hypothesis is that slug caterpillars without spines may be physiologically more suited to dry environments, such as deserts, seasonal savannas, and alpine woodlands (Leuschner, 2000). According to previous studies (Battisti et al., 2011; Cock et al., 1987; Epstein et al., 1999; Hossler, 2010; Kano, 1977; Kawamoto & Kumada, 1984), spines on nettle caterpillars consist of multiple cells, and spines are usually arranged on tubercles. Slug caterpillars with spines on tubercles have higher surface area to volume ratios than slug caterpillars without spines and tubercles. Surface area to volume ratios may influence water balance in ectotherms (Ashton, 2002; Bidau & Marti, 2008). For example, the tropical rain frog, *Eleutherodactylus coqui*, reduces water loss by adjusting posture and activity to control the exposed surface area (Pough, Taigen, Stewart, & Brussard, 1983; Vitt & Caldwell, 2013). By analogy, slug caterpillars without spines with lower surface area to volume ratios may be more suited to dry environments. In a previous study, it has been observed that nettle caterpillars are distributed more in tropical areas and gelatine caterpillars are distributed more in temperate areas (Zaspel et al., 2016).

In addition to adaptation to similar local environments, because genetic or developmental constraints limit the generation of phenotypic variations (Brakefield, 2006; Hall, 2007; Wake et al., 2011), the reappearance of similar features in organisms may result from different selective pressures (Hall, 2007). For example, pelvic reduction in stickleback populations, which are sympatric with various fish and bird predators, may be triggered by low calcium ion concentration (Giles, 1983); in Paxton Lake with a high calcium ion level and in some Alaskan Lakes with lack of native predatory fishes, stickleback populations have similar pelvic vestiges (Bell et al., 1985; Larson, 1976). Therefore, homoplasy of pelvic reduction in sticklebacks is more likely to be caused by different selective pressures, low calcium ion concentration and lack of native predatory fishes, in different lakes (Bell, 1987). Furthermore, homoplasy is common with reduced characters especially for complex characters, which may have low probability of origin but can be lost or reduced by the action of a few genes (Culver & Pipan, 2016; Cunningham, Omland, & Oakley, 1998; Maddison, 1994; Sackton et al., 2019). In this study, the larvae of *Caiella pygmy* occur in montane areas (above 2500 m) in winter and spring, whereas those of *Ecnomoctena brachyopa* and *Pseudanapaea transvestita* are distributed in relatively dry areas of Australia. Thus, loss of spine may be evolved to response to different environments because of genetic constraints.

Finally, spine loss in slug caterpillars may be just fixed by random genetic drift, especially at the ancestral state in lineage 3, because most of these species with spine loss in late instars (character state B) are sympatric with most species from Asia in lineage 4‐6 in which spines are present in late instars (character state A). Hence, homoplasy of spine loss in the Limacodidae may be the result of one or more processes, including adaptation to similar local environments, shared constraints, and random genetic drift.

### 4.2 Systematic considerations

In the inferred phylogenetic tree of the Limacodidae, we identified six lineages (Figure 2). Lineage 1 contains *Trichogyia limacodiformis*, *Microleon longipalpis* and sp. 1, a clade which had not been identified in previous phylogenetic studies of the Limacodidae. Interestingly, this clade was recovered relatively deep in our phylogeny, being sister to lineages 2 and 3. Lineage 1 shares several morphological characters, such as small body size (forewing length <10 mm) and character state A. The structure of the spine in lineage 1 is the same as that in lineages 4–6, which is formed by trichogen cells that line up with the epidermal cells (Kawamoto & Kumada, 1984), although the numbers of spines on each segment (Figure 1d,h) are fewer than those in lineages 4–6 (Figure 1b,c,f,g).

In lineage 2, three taxa comprise a monophyletic group that is characterized by hairy monkey slug caterpillars (character state D). The clade includes *Isochaetes* sp. and *Phrixolepia inouei*, which emerged as sister taxa. The geographical distribution of *Isochaetes* is in eastern North America, Central America, and northern South America, whereas the distribution of *Phrixolepia* is mainly in eastern Asia (Ratnasingham & Hebert, 2007). The disjunction between North America and eastern Asia has been reported for many animal and plant taxa (Espeland et al.^2015^; Nordlander, Liu, & Ronquist, 1996; Peña, Nylin, Freitas, & Wahlberg, 2010; Tiffney, 1985; Wen, 1999). Thus, *Isochaetes* and *Phrixolepia* may provide another example of dispersal (and extinction) through the Bering land bridge that formerly connected North America with Eurasia.

The large clade including lineages 4–6 containing most of the nettle caterpillars with spines present after second instar is phylogenetically equivalent to the “nettle” clade identified by Zaspel et al. (2016). In both clades, most, if not all, species fast in the first instar, which is that the first instars do not feed on the host plant and then they quickly molt to the second instar. Interestingly, in our study this clade included *Caiella pygmy*, *Pseudanapaea transvestita*, and *Ecnomoctena brachyopa* in lineage 6 in which there were transitions from late instars with spines to late instars with spines lost or reduced. From the rearing experience, *Caiella pygmy* and *Pseudanapaea transvestita* still retain the fasting behavior in the first instar. However, we do not know if fasting in the first instar applies to *Ecnomoctena brachyopa*.

Within lineage 6, we found that the genus *Parasa* is not monophyletic because of inclusion of the species *Caiella pygmy*. Solovyev (2010) originally described the species *pygmy* in the genus *Parasa*. Later, Solovyev (2014) revised *Parasa* and transferred *P. pygmy* to his newly described genus *Caiella* based on adult forewing pattern and the reduced scoli in mature larvae. However, our phylogenetic results indicate that *Caiella pygmy* renders *Parasa* paraphyletic. Further, the character reconstruction in this study revealed that reduced scoli in late instar larvae is the result of homoplasy and should not be regarded as an autapomorphy to diagnose the genus. Hence, either the genus *Caiella* needs to be synonymized with *Parasa* or many of the subgroups within *Parasa* need to be elevated to monophyletic genera. Since *Parasa* currently comprises about 240 species, we suggest the monophyly of the genus needs further investigation until any taxonomic change is made.

With the exception of *Chalcocelis albiguttatus*, all other taxa from Australia (seven species representing six genera) comprised a monophyletic group within lineage 6 (Figure 3: Node 5). Although the clade was not strongly supported, it may be improved by greater taxon sampling of the fauna of the continent. The topology and relative branch lengths indicate that most limacodids in Australia evolved relatively recently. Moreover, the Australian lineage is nested within a set of predominantly Asia lineages (lineages 4–6), which suggests that the origin of these limacodids is not in Australia. Further taxon sampling of the family and divergence times using a molecular clock are needed to estimate deeper biogeographic patterns to test this hypothesis.

## CONFLICT OF INTEREST

None declared.

## Data Availability

The data that support the findings of this study are openly available in GenBank at https://www.ncbi.nlm.nih.gov/genbank/, accession numbers in Table 1.
